# A Lightweight Internet Sharing Scheme for Sectional Medical Images according to Existing Hospital Network Facilities and Basic Information Security Rules

**DOI:** 10.1155/2020/8838390

**Published:** 2020-12-04

**Authors:** Liang Qiao, Hao Wu, Yi Wu, Wenjing Wu, Jingyi Yang, Yongjian Nian, Mingsheng Chen, Sen Bai, Hao Huang, Mingguo Qiu

**Affiliations:** ^1^College of Biomedical Engineering and Imaging Medicine, Army Medical University, Chongqing, China; ^2^Information Technology Center, Army Medical University, Chongqing, China; ^3^Department of Radiology, Southwest Hospital, Army Medical University, Chongqing, China; ^4^College of Big Data and Artificial Intelligence, Chongqing Institute of Engineering, Chongqing, China; ^5^Department of Information, Army Medical Center, Chongqing, China

## Abstract

**Background:**

With the outbreak of COVID-19, large-scale telemedicine applications can play an important role in the epidemic areas or less developed areas. However, the transmission of hundreds of megabytes of Sectional Medical Images (SMIs) from hospital's Intranet to the Internet has the problems of efficiency, cost, and security. This article proposes a novel lightweight sharing scheme for permitting Internet users to quickly and safely access the SMIs from a hospital using an Internet computer anywhere but without relying on a virtual private network or another complex deployment.

**Methods:**

A four-level endpoint network penetration scheme based on the existing hospital network facilities and information security rules was proposed to realize the secure and lightweight sharing of SMIs over the Internet. A “Master-Slave” interaction to the interactive characteristics of multiplanar reconstruction and maximum/minimum/average intensity projection was designed to enhance the user experience. Finally, a prototype system was established.

**Results:**

When accessing SMIs with a data size ranging from 251.6 to 307.04 MB with 200 kBps client bandwidth (extreme test), the network response time to each interactive request remained at approximately 1 s, the original SMIs were kept in the hospital, and the deployment did not require a complex process; the imaging quality and interactive experience were recognized by radiologists.

**Conclusions:**

This solution could serve Internet medicine at a low cost and may promote the diversified development of mobile medical technology. Under the current COVID-19 epidemic situation, we expect that it could play a low-cost and high-efficiency role in remote emergency support.

## 1. Introduction

Sectional Medical Images (SMIs), including CT and MRI, are the main objects of medical image diagnosis at present [1, 2]. They can be used to obtain any viewing angle via multiplanar reconstruction (MPR) and maximum/minimum/average intensity projection (MIP/MinIP/AVG or MIPs for short) [3]. They are of great significance in medical diagnosis and treatment [4]. However, since every interaction of MPR and MIPs is based on the reconstruction of the raw data, this means that hundreds of megabytes of SMIs data have to be transferred to the client, resulting in a higher requirement for network transmission and a higher risk of a data leakage; therefore, the scope of their use is too limited to apply to the Internet [5].

Taking the COVID-19 outbreak as an example, due to the community segregation policies implemented in many countries and a lack of radiologists in the epidemic areas or less developed areas [6], hospitals requiring remote support face two prominent problems. (1) Network transmission bottlenecks: assuming that every radiologist diagnoses 20 CT reports a day while dozens of radiologists can work over the Internet, how can the hundreds of gigabytes of data be transmitted quickly over the Internet? Especially during the epidemic, large-scale online work and online learning have made network facilities vulnerable [7]. (2) Security risk of data leakage: in terms of hospital information security rules, the PACS (picture archiving and communication system), the RIS (radiology information system), and other hospital Intranet systems [8] are strictly isolated from the Internet; how can we ensure that the original image data could be used over the Internet but kept in the hospital?

Regarding problem (1), in the preliminary work, we used a remote rendering scheme to solve the transmission pressure on the network to achieve the purpose of lightweight sharing. The core concept is to put the reconstruction work on the server side to respond to client requests in real time and to project the final reconstruction results back to the client for presentation. The client is just a window that submits instructions and displays results [9, 10]. Although the network transmission bottleneck and the risk of data leakage were both reduced, the *last mile problem* from Intranet PACS to the Internet and the *interactive experience* for MPR or MIPs reconstruction have not been considered. Regarding problem (2), it is difficult to break through the basic hospital information security rules of network isolation only by improving the data transmission encryption algorithms [11–14]; moreover, the mode of regional health service via a VPN between designated hospitals has difficulty meeting the needs of open multipoint access in the Internet environment [15]. Although there are many “hospital-VPN-cloud-Internet client” schemes that combine the advantages of data encryption and private network [16], the security issues of the large-scale uploading of the original image data to the Internet cloud has always been controversial, and the security approval procedures are complex and costly.

In fact, in the face of rigid demand such as online appointments or online inquiry for diagnostic reports, which must retrieve the desired data from the hospital's Intranet via the Internet, there are mature schemes that exist in the security rules, such as “firewall & front-end computer”, “gateway & front-end computer” or a combination of both [17]. The front-end computer is the only normalization facility with limited cross network authority in a hospital to achieve the monitored data flow via deployed customized applications.

With the help of the above mature scheme and our early remote rendering work, this paper proposes a four-level endpoint network penetration scheme of “PACS-Automatic Image Intermediary Agent (AIIA)-front-end computer-Internet client,” which relies on the existing hospital network facilities and security rules, to realize the safe and lightweight sharing of huge SMI files over the Internet. Furthermore, to address the problem of nonnative interactive experiences in the current remote rendering schemes [18–22], a “Master-Slave” two-channel interactive method for MPR and MIPs reconstruction was designed. Finally, a prototype system was formed and tested to realize a diagnostic assistance function for doctors outside the hospital with a lightweight client hardware and network environment. It is expected to play a role in the COVID-19 outbreak.

## 2. Methods

### 2.1. Basic Architecture

**Figure 1:**
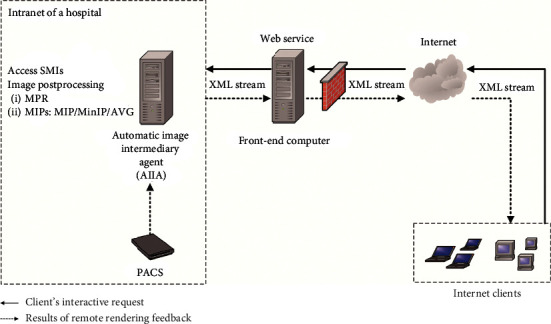
Basic architecture of the network penetration scheme of “PACS-AIIA-front-end computer-Internet client.”

Figure 1 shows the “PACS-AIIA-front-end computer-Internet client” four-level endpoint network penetration scheme designed in this paper. PACS is the actual storage location of the SMIs in the hospital Intranet. AIIA is the only hardware facility that needs to be deployed in the hospital Intranet as the remote rendering server and is responsible for accessing PACS data, performing image postprocessing (MPR, MIPs, etc.) according to the interactive requests from Internet clients, and feeding back the two-dimensional processed pictures to the front-end computer. The front-end computer is an existing facility of the hospital that bridges the internal and external networks to transmit the requests from the Internet client to the AIIA and transmit the two-dimensional processed pictures from the AIIA to the Internet client. Internet clients can access the front-end computer via ordinary Internet equipment from anywhere and carry out a remote rendering scheme for image postprocessing.

Figure 1 is also a data flow diagram based on the internal and external network interfaces of the front-end computer. To meet the rigid demands of data exchange and ensure information security as much as possible, the information security rules strictly limit the communication ports of the front-end computer. Generally, only port 80 is open, and the HTTP communication mode is used to bridge the data. Therefore, to avoid the reconstruction of communication rules and additional complex security approval procedures, we used “web service + XML” technology to connect the AIIA to feed data back over the Intranet and fulfill the client interactive requests over the Internet.

A web service can be simply understood as a web application based on HTTP communication [23]. In this scheme, one was deployed as a web server on the front-end computer, as shown in Figure 1. The submitters both from the Internet client and AIIA only need to call the web service interface to transmit the agreed XML stream to the web server [24], and the XML could be analyzed by the web server to complete the data exchange.

The core work includes the design of the XML data exchange interface model and the design of the overall architecture integrated with the “Master-Slave” dual-channel interaction.

### 2.2. Design of XML Data Exchange Interface Model

An XML (extensible markup language) [25] document was used to accurately record the interactive requests of the Internet client and the processing results from the AIIA; therefore, the model was designed to cover the following four types.

1. Interactive request: used to accurately record the final interactive requests of the Internet client on the SMIs, mainly focusing on the interaction of MPR and MIPs
2. Interactive object: includes SMI objects, times, operators, and so forth
3. Blocking feedback: since the image postprocessing of the AIIA needs a certain amount of operating time, the XML was required to record and transfer the data processing status of each pipeline to maintain the interactive order
4. Pipeline management: used for multiple clients sending requests to the AIIA to view and process their own SMIs at the same time

According to the rules of XML syntax, we extracted the structural characteristics of the interactive behavior and business responses of clients and built a unified storage model covering 11 XML elements. These are shown in Table 1.

**Figure 2:**
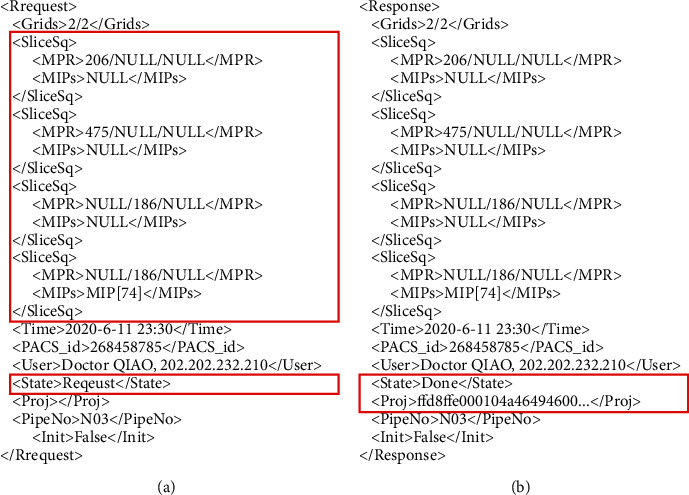
XML documents generated after a client interaction and after an AIIA response: (a) request from Internet client and (b) response to Internet client.

Figure 2 shows two examples of XML that were, respectively, generated by an Internet client after an interactive operation and by the AIIA after responding.

**Figure 3:**
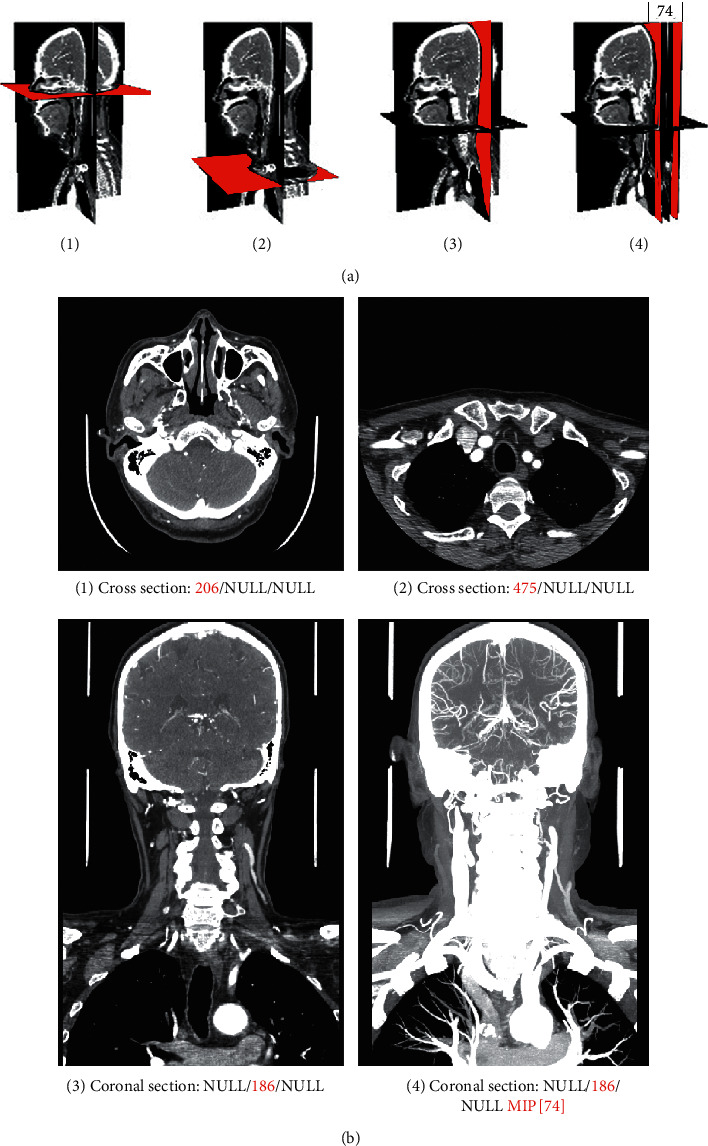
The section view corresponding to the XML document example in Figure 2.

Figure 2(a) described *a* <Request> message generated from a client. This XML requests that the Intranet AIIA extracts a CT dataset with ID number 268458785 from the PACS system and generates a 2D digital image film that maintains the original quality. The film consists of 4 MPR sections in the 2 × 2 split screen mode, including 2 cross sections and 2 coronal sections. The section locations are shown using red marks in Figure 3(a), of which the fourth coronal image needs an MIP projection. The request was initiated by Dr. Qiao at 23 : 30 on June 11, 2020. The pipeline number of the client was N03.

Figure 2(b) shows the <Response> message from the Intranet AIIA. Corresponding to the <request> message, the <Proj> and <State> tags were added. <Proj> was used to record the hex stream text of the zip file for the projection data from the AIIA. The message presented in the client is shown in Figure 3(b).

### 2.3. Design of the Overall Architecture Integrated with the Master*-*Slave Dual-Channel Interaction

**Figure 4:**
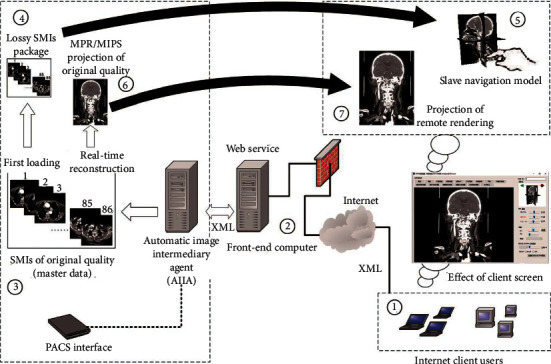
The overall architecture of the network penetration scheme integrated with the Master-Slave dual-channel interaction.

In traditional remote rendering, to improve the user experience, the trajectory of a single interactive operation from the client was often divided into several request points to request continuous responses from the server [18, 26]. However, the traditional method has resulted in huge pressure on both the server response and the network feedback. Based on the basic data penetration architecture in Figure 1, this paper further adopted the “Master-Slave” two-channel interaction [9] to solve the interactive experience problem of remote rendering technology. The overall structure is shown in Figure 4.

Figure 4 includes the PACS interface, AIIA, hospital front-end computer, and Internet client. The core is the navigation interaction of the “Slave” model by the client at zone ⑤ and the high-quality processing and projection of the “Master” data by the AIIA at zone ③. The AIIA is used as the central server for remote rendering. Through the front-end computer, it receives rendering requests from the Internet client and then automatically retrieves the corresponding original SMI data from the PACS interface to form the “Master” data. According to the simulated navigation of the “Slave” model on the client side, the high-quality 2D projection of the reconstruction of the MPR and MIPs that users truly care about can be completed accurately, and then it can be sent back to the Internet client through the front-end computer. Regarding the feedback results, the Internet client can handle local processing such as size measurement, window-level rescaling [27], and pseudo-coloring.

The processing steps are as follows.

For the first loading, we do the following.

  Step 1. The Internet client (at zone ①) submits the XML of the ID number of SMIs to the front-end computer of the hospital (at ②) through the web service, and the front-end computer transfers the XML to the AIIA workstation in the hospital Intranet. The AIIA parses the XML and loads the corresponding SMIs into the memory from PACS (at ③).
   Step 2. The resident process on the AIIA will automatically shrink the SMI data of each frame to a resolution of 64 × 64, clear the tag information related to the patient's privacy in the DICOM, to form a “lossy SMIs package” (at ④), which is packaged as a zip file, fed back to the front-end computer through XML format, and passed to the Internet client.
  Step 3. The client receives the zip file of the lossy SMI package, decompresses the zip, and rebuilds the “Slave” navigation model (at ⑤) to facilitate user interaction.

In Step 2, the data size of “loss SMIs package” is greatly reduced from the original SMIs, it can be quickly calculated according to the following formula:

(1) $$\begin{array}{c} {\text{ds}}_{\text{slave}}=\sum_{i=0}^{\text{frames}}\text{Res}N^{2}\times \text{bitdepth}. \end{array}$$

Here, ds_slave_ is the data size of “loss SMIs package” and the unit is KB, frames is the total number of frames of the original SMIs, Res*N* is the destination resolution of each frame to be compressed (default is 64), and bitdepth is pixel depth of original SMIs which can be read from DICOM tag of “(0028,0100) US Bits Allocated”.

For the “Master-Slave” interaction after the first loading, we perform the following:

  Step 1. The client simulates the MPR, MIP, and other operations through the “Slave” navigation model (at ⑤) and submits the XML document generated when the operation is completed to the hospital front-end computer (at ②), through a web service.
  Step 2. The front-end computer transfers the XML to the AIIA, which processes the corresponding “Master” data (at ③) loaded into memory in the early stage according to the client's request, and projects it into a 2D DICOM file that has removed the patient's privacy data (at ⑥).
  Step 3. Then, the 2D DICOM file is packaged as a zip file, fed back to the front-end computer through XML format, and passed to the Internet client for the final presentation (at ⑦).

If there is another Master-Slave interaction, return to Step 1.

### 2.4. Prototype System Implementation

The prototype system is divided into three parts: the AIIA hardware and its resident process software, the front-end computer hardware and its web service listening process software, and the Internet client processing software. Here, the front-end computer hardware is an existing utility of the hospital and is used only as a web service interface process to transfer XML data flows without additional hardware deployment. The AIIA can select a general image workstation in the radiology department to access the Intranet PACS, and its hardware configuration and scale are determined by the scale of the external requests undertaken.

The software development includes three main parts: the AIIA listening and image postprocessing, the web service on the front-end computer, and the interaction and DICOM browsing on the client side. All of them are based on Visual Studio 2008 development platform with VTK component for *Slave* navigation [28] and the ClearCanvas component for DICOM tagging [29]. Each part has a real-time listening process to ensure the automatic processing of the whole process.

This architecture is open-designed. If a mature third-party commercial image workstation system provides an image processing command interface, it can also be deployed on the AIIA and connect to the architecture designed in this paper.

## 3. Results

### 3.1. Operation Sketch and Interactive Experience for Internet Clients

**Figure 5:**
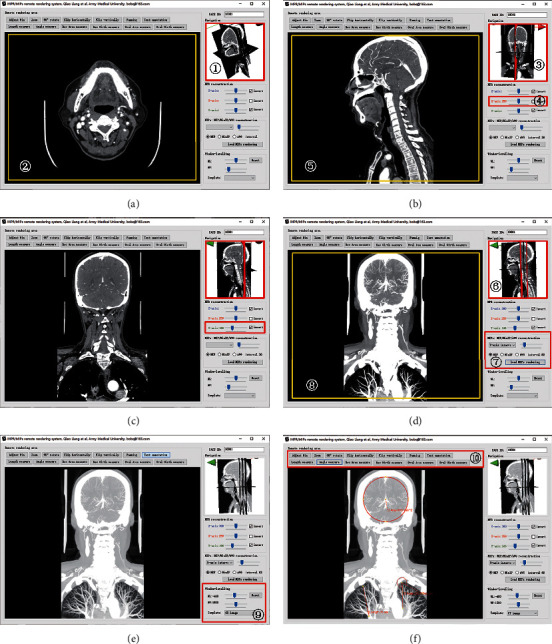
Screenshot of the client operation sketch of the prototype software according to the architecture of this paper (the corresponding demo video is shown in the supplementary materials). (a) Initial stage. (b) MPR interactive perspective (sagittal plane). (c) MPR interactive perspective (cross section). (d) Set MIP interval (cross section). (e) Local window-levelling based on D (from chest window to lung window). (f) Carry out size measurement and other operations based on E.

Figure 5(a) shows the initial state of the “Slave” model (at zone ①, 6.99 MB of reconstruction data on the client side) and the remote rendered high-quality 2D DICOM projection (at zone ②, 307.04 MB of original SMIs data on the server side).  Figure 5(b) shows the use of a mouse or touch-screen gesture to change the viewing angle of the “Slave” model (at zone ③) and the use of a mouse wheel or slider to select the sagittal section (at zone ④). When the interactive operation stopped, the software automatically submits the interactive request to the server, and the 2D DICOM projection from the “Master” data (at zone ⑤) is obtained.  Figure 5(c) shows the use of the same method as Figure 5(b) to browse the coronal section.  Figure 5(d) shows the dynamic setting of the MIP reconstruction interval (at zone ⑥) on the “Slave” model through the MIPs control panel (at zone ⑦); subsequently, the “load MIPs rendering” button was clicked to submit the interactive request to the server. The DICOM projection from the “Master” data (at zone ⑧) is obtained. Figures 5(d) and 5(e) show local window-level rescaling through a window-level panel (at zone ⑨), and Figure 5(f) shows further use of the local tool kit (at zone ⑩) for size measurement and other operations. The processes shown in Figures 5(d) and 5(f) do not require interaction with the server. The corresponding demo video is shown in the supplementary materials.

Furthermore, five radiologists were invited to test the prototype software according to the behavior in Figure 5 using 4G Internet mobile hotspot access. A questionnaire using a 5-point Likert scale was designed for participants, and the feedback results are shown in Table 2.

All of the respondents agreed that this is an interesting and meaningful method that can quickly access the internal data of the remote hospital and that the imaging quality is no different from that of a traditional local image workstation. It is meaningful to transform the prototype system to the business system for practical diagnosis.

Compared with the popular remote rendering method of “continuous request-continuous response” [18], our method adopted the “Master-Slave” interaction mode to enhance the user experience. Although [20–22] mentioned that the network congestion caused by “continuous request-continuous response” could be improved by using video-compressed transmission [20], variable resolution transmission [21], tile-based transmission [22], and other methods, the server load pressure increased significantly. For example, if wanting the rendering capacity of the client to reach 30 fps, the server will be required to provide 30 continuous responses and 30 image compressions and network transmissions within 1 second. In our team's preliminary work in [9], through low-quality 3D navigation, we avoided continuous server response and network load pressure in the interaction process, but that work was only for 3D volume rendering operations. This paper has designed a navigation mode for MPR and MIPs to improve interaction efficiency, which has been recognized by five radiologists.

### 3.2. Performance Testing and Information Security

To facilitate the quantitative test, the prototype system's deployment was carried out using the campus LAN of the Army Medical University. The detailed parameters of the equipment are as follows.

#### 3.2.1. Front-End Computer

E5-2630 2 × 2.4 GHz CPU, 8 GB of RAM, 1000 Mbps LAN environment, and 500 Mbps Internet environment.

#### 3.2.2. AIIA

E5-2603 2 × 1.8 GHz CPU, 16 GB of RAM, and 1000 Mbps LAN environment.

#### 3.2.3. Internet Client

Three PCs (Win 7, 4 GB of RAM) are located on a 2 Mbps mobile network, each of which was limited to a 200 kBps bandwidth by a 360 security firewall for extreme testing [30]. Three junior students majoring in biomedical engineering were invited to test and quantize the network load and response time.

#### 3.2.4. Testing Data

Three groups of SMIs from the CT Equipment include (1) head and neck CT with 610 slices at a resolution of 512 × 512, totaling 307.04 MB; (2) trunk CT with 609 slices 512 × 512, totaling 306.5 MB; and (3) thorax and mandible CT with 500 slices 512 × 512, totaling 251.6 MB. According to the generation rules of the lossy SMIs package at ③, ④, and ⑤ in Figure 4, the actual data sizes of the lossy SMIs package from each testing data are shown in Table 3 and are consistent with the theoretical value of formula (1).

The operating behavior of each client included the first loading, MPR browsing, and MIPs browsing of testing data (1), (2), and (3). The tests lasted for one hour, focusing on monitoring the network traffic and response time. Three students were required to independently operate the clients within the same time range. The results are shown in Table 4.

Table 4 presents the average response time of each client for the three types of interactive behaviors with a 200 kBps Internet bandwidth. The total response time includes the AIIA image processing delay and the “PACS-AIIA-front-end computer-Internet client” network delay. The AIIA image processing delay can be reduced by the improving image processing algorithm or using better graphics products, and the network delay is the focus of this paper, which corresponds to the amount of data transferred.

While a user accesses a group of SMIs, after an average of 23.96 seconds for the first loading (including approximately 18 seconds for Internet transmission with a bandwidth of 200 kBps), the “Slave” model can be built in the client for the MPR and MIPs interactive navigation. The final original-quality projections of the MPR and MIPs for each request take 1.63 seconds and 1.90 seconds, respectively. If we deduct the image processing delay, which has huge improvement space, the network times are only 1.12 seconds and 1.10 seconds, respectively. If the 200 kBps bandwidth limit is removed, the response speed will be far less than 1 second, and this can solve the bottleneck problem of network transmission.

Regarding information security, three engineers from the hospital information department were invited to search the client disk and memory for data leakage security issues. It was found that the “Slave” data and some present projection results were stored in the temporary folder of the local client and would be deleted automatically after the software exits. Even if the temporary files were saved in private, the 64 × 64 resolution of each frame of “Slave” data could only be used for interactive navigation. Although the projection results were in DICOM format, the patient's private information was discarded when the projection was created in the AIIA and could not be obtained by the client; moreover, the original data was always kept in the Intranet of the hospital.

## 4. Discussion

This paper proposed a four-level endpoint network penetration scheme for SMIs based on existing hospital network facilities and hospital information security rules that enable authorized users to access high-quality images in real time on the Internet. Furthermore, we design the “Master-Slave” two-channel interaction based on the characteristics of the MPR/MIPs interaction to enhance the user experience and get desired results. Compared with the popular “hospital-VPN-cloud-Internet client” scheme, our method does not require complex approval procedures and line construction, especially suitable for emergency deployment.

For Internet users, the prototype system can support doctors outside the hospital. Doctors can use a normal computer connected to the Internet anywhere to freely operate the image data authorized by the hospital with equivalent quality. For hospital managers, the “remote rendering + four-level endpoint network penetration” mode could ensure that outside doctors cannot obtain the original data and private patient information and ensure safety. Regarding the system deployment, this architecture has the advantages of being flexible, simple, and fast, which allows outside doctors to assist in image diagnosis at a low cost. Depending on the number of Internet users (user scale), the AIIA can be deployed on an existing image workstation or on an independent server cluster; the “AIIA-user scale” relationship model will be the focus of our next study.

## 5. Conclusion

We noticed that there are a lot of studies focusing on new technologies and clinical practices in the telemedicine field, but few studies focu on the basic practice of hospital data flow. Due to the technical barriers between clinical staff and IT enterprises, the deployment and promotion of a new cross network system are always costly. With the outbreak of COVID-19, some hospitals in the epidemic area are desperate to establish larger teleconsultation rooms, to upgrade VPN or another network with constant debates on security rules; however, some simple but useful technical aids have been ignored. Therefore, this paper proposed a lightweight and rapid SMIs sharing scheme that is easy to deploy and built a prototype system for performance demonstration. This scheme can provide telemedicine and Internet medicine service at a low cost, which may promote the diversified development of mobile medical technology. We expect that this scheme could be known by more medical staff in the epidemic area and play a role in low-cost and high-efficiency remote emergency support.

## Figures and Tables

**Table 1:** XML format interface model.

Tag name	Demonstration	Data flows	Data type	Event type
*Grids*	For a split screen, output *m* × *n* section pictures at a time. Format: m/*n*	Client initiated	Integer	Interactive request
*SliceSq*	For a slice sequence, multiple sets of MPR/MIPs child nodes could be embedded	Client initiated	With child nodes	
*MPR*	Used to represent the positions of the coronal plane, cross section, and sagittal plane, respectively. Format: *X*/*Y*/*Z*, for example, 20/NULL/NULL represents getting a coronal plane whose coordinate is (20,0,0).	Client initiated	Integer	
*MIPs*	Includes four types: MIP, MinIP, AVG, and NULL. If the MIPs are not NULL, format: MIPs type [interval]	Client initiated	Text	
*Time*	Occurrence time of client interaction	Client initiated	DateTime	Interactive object
*PACS_id*	Unique identification number of a group of SMIs from PACS	Client initiated	Text	
*User*	Account and IP address of client	Client initiated	Text	
*State*	Response status of the AIIA, including *Request*, *Loading*, *Processing,* and *Done*	AIIA response	Text	Blocking feedback
*Proj*	Projection (2D pictures) from the AIIA rendering, recorded with hex stream text after zip packing	AIIA response	Text	
*PipeNo*	For a data flow pipeline, a group of SMIs could be independently operated by multiple clients with different pipelines.	Client initiated	Text	Pipeline management
*Init*	Recorded as true or false depending on whether it is the first requested operation of the dataset by the client.	Client initiated	Boolean	

**Table 2:** Statistical results of the questionnaire on interactive experience and imaging quality using a 5-point Likert scale.

Focus	Subject	Five-scale				
		Very much agree	Agree	Not sure	Disagree	Completely disagree
Interactive experience	The interactive operation is accurate.	5				
	It is simple and easy to use without training.	5				
	It gives a good user experience similar to the native application.	4	1			
	The latency time for the final high-quality image is acceptable	5				
Imaging quality	The *Slave* model could meet the navigation demands.	5				
	The 2D projection image quality from the *Master* volume is no different from that of a traditional local workstation.	5				
Others	It is meaningful to transform the prototype system to business system for practical diagnosis.	5				

**Table 3:** The data size of the lossy SMIs packages (for the *Slave* dataset) for each testing data.

Test data	Original SMIs		Lossy SMIs package (for *Slave* dataset)		
	Resolution	Data size (MB)	Resolution	Data size (MB)	Zip package (MB)
Test data (1)	512 × 512 × 610	307.04	64 × 64 × 495	6.99	**3.74**
Test data (2)	512 × 512 × 609	306.5	64 × 64 × 609	6.57	**3.71**
Test data (3)	512 × 512 × 500	251.6	64 × 64 × 500	5.39	**3.04**
Average				6.32	**3.50**

**Table 4:** The average response time and amount of data transferred of each client for the three types of interactive behaviors under a 200 kBps Internet bandwidth for the extreme test.

Internet device	First loading (average)			MPR projection (average)			MIPs projection (average)		
	Amount of data transferred (MB)	Response time (seconds)		Amount of data transferred (KB)	Response time (seconds)		Amount of data transferred (KB)	Response time (seconds)	
		Total	Network		Total	Network		Total	Network
Client 1	3.54	23.89	**21.34**	229.60	1.58	**1.15**	237.67	2.10	**1.19**
Client 2	3.55	24.11	**21.42**	237.63	1.85	**1.19**	191.51	1.63	**0.96**
Client 3	3.52	23.89	**21.46**	206.35	1.46	**1.03**	229.09	1.97	**1.14**
Average	3.54	23.96	**21.41**	224.52	1.63	**1.12**	219.42	1.90	**1.10**

## Data Availability

The data used to support the findings of the study are available from the corresponding author upon request.

## Supplementary Materials

Supplementary Materials A video of the prototype system demonstration can be downloaded from supplementary materials.
