# Supplementary material for: A Lightweight Internet Sharing Scheme for Sectional Medical Images according to Existing Hospital Network Facilities and Basic Information Security Rules
Source: J Healthc Eng. 2020 Dec 4;2020:8838390. doi: 10.1155/2020/8838390 (PMC7737442; doi:10.1155/2020/8838390)
Supplement: Supplementary Materials — A video of the prototype system demonstration can be downloaded from supplementary materials. [file 8838390.f1.zip › 8838390.f1/Introduction to the video of prototype system demonstration.pdf]

Introduction to the video of prototype system demonstration

for lightweight accessing of Sectional Medical Images from *hospital Intranet* by Internet users

The final high quality image of MPR and MIPs was accessed by remote rendering, the interactions of *Slave* model, window-levelling and dimension measurement were run on the client side.

Detail:

(1) First loading

i.	Open the application	00:00:07
ii.	Enter the ID number of PACS, wait the *Slave* model loading	00:00:12

(2) Interactive behavior

i.	MPR browsing: *Slave* model interaction and requesting the remote rendering	00:00:21
ii.	Window adjustment on the local client side	00:00:41
iii.	MPR browsing: *Slave* model interaction and requesting the remote rendering	00:00:49
iv.	MIP browsing: setting the MIP interval area on the *Slave* model and requesting the remote rendering	00:00:58
v.	Window adjustment on the local client side	00:01:19
vi.	Dimension measurement on the local client side	00:01:28
